# Polyethylene degradation and assimilation by the marine yeast *Rhodotorula mucilaginosa*

**DOI:** 10.1038/s43705-023-00267-z

**Published:** 2023-07-10

**Authors:** Annika Vaksmaa, Lubos Polerecky, Nina Dombrowski, Michiel V. M. Kienhuis, Ilsa Posthuma, Jan Gerritse, Teun Boekhout, Helge Niemann

**Affiliations:** 1 Department of Marine Microbiology and Biogeochemistry, NIOZ Royal Netherlands Institute for Sea Research, Texel, The Netherlands; 2 Department of Earth Sciences, Faculty of Geosciences, Utrecht University, Utrecht, The Netherlands; 3 Deltares, Unit Subsurface and Groundwater Systems, Utrecht, The Netherlands; 4 Westerdijk Fungal Biodiversity Institute, Utrecht, The Netherlands; 5 Institute of Biodiversity and Ecosystem Dynamics (IBED), University of Amsterdam, Amsterdam, The Netherlands; 6 College of Science, King Saud University, Riyadh, Saudi Arabia

**Keywords:** Microbiology, Environmental sciences

## Abstract

Ocean plastic pollution is a severe environmental problem but most of the plastic that has been released to the ocean since the 1950s is unaccounted for. Although fungal degradation of marine plastics has been suggested as a potential sink mechanism, unambiguous proof of plastic degradation by marine fungi, or other microbes, is scarce. Here we applied stable isotope tracing assays with ^13^C-labeled polyethylene to measure biodegradation rates and to trace the incorporation of plastic-derived carbon into individual cells of the yeast *Rhodotorula mucilaginosa*, which we isolated from the marine environment. ^13^C accumulation in the CO_2_ pool during 5-day incubation experiments with *R. mucilaginosa* and UV-irradiated ^13^C-labeled polyethylene as a sole energy and carbon source translated to degradation rates of 3.8% yr^–1^ of the initially added substrate. Furthermore, nanoSIMS measurements revealed substantial incorporation of polyethylene-derived carbon into fungal biomass. Our results demonstrate the potential of *R. mucilaginosa* to mineralize and assimilate carbon from plastics and suggest that fungal plastic degradation may be an important sink for polyethylene litter in the marine environment.

## Introduction

Ocean plastic pollution is an environmental problem of exponentially increasing magnitude [1–6]. However, uncertainty exists on the importance of individual pathways through which plastic is transported from land to the ocean [7–11], how much plastic remains in coastal areas [12], the open ocean surface [13, 14], the mid-water column [15–17], or sinks to the ocean floor [18, 19]. Current estimates of the total amount of plastic at the ocean surface only account for less than 1% of the estimated amount of all plastics that have ever been released into the sea [20, 21]. Thus, an unknown sink mechanism apparently removes detectable plastic debris from the ocean surface [2, 21]. This might be an abiotic process, such as fragmentation to micro and nanoscale particles [21] or photodegradation, which destabilizes the polymer structure [22] and causes leaching of dissolved organic carbon [3, 23–25]. In addition, the removal mechanism can also be biotic mineralization, mediated by microbes such as bacteria, archaea, or fungi. Plastic degradation by bacteria has been well documented for terrestrial and marine environments. For example, the terrestrial bacterium *Ideonella sakaiensis* hydrolyzes polyethylene terephthalate [26–28], while *Rhodococcus ruber* strain C208 has been found to degrade polyethylene (PE) and polystyrene (PS) [29–31]. In addition, the marine species *Bacillus sphericus* and *Bacillus cereus* have been shown to degrade polyethylene [32]. In contrast to bacteria, our knowledge of the potential role of fungi-mediated plastic degradation is in its infancy, especially in the marine environment. Due to their genetic and metabolic capabilities, fungi are best known as the main degraders of natural polymers such as wood, plants, cellulose, and lignin. Furthermore, fungi are efficient degraders of various complex hydrocarbons, including polycyclic aromatic hydrocarbons [33], oil, and alkanes [34, 35]—i.e., compounds that to some extent chemically resemble plastic. Fungi harbor powerful enzymatic machineries comprising, for example, manganese peroxidases, lignin peroxidases, and laccases [36–38], which have also been linked to plastic degradation [39, 40]. With respect to plastic degradation, multiple *Aspergillus* and *Penicillium* species, isolated from soils and gut microbiomes, were shown to degrade polyethylene [41–46]. Though fungi are common plastic colonizers in the ocean [47–51], only two species, *Zalerion maritimum* [52] and *Alternaria alternata* [53], have been identified as polyethylene degraders in the marine realm.

A general problem in measuring microbial plastic degradation and comparing results from different studies stems from methodological challenges and limitations. The chemical configuration of the used polymers, for example, the degree of crosslinking, crystallinity, and the addition of additives, as well as the size of the plastic particles and environmental/incubation conditions, likely affect microbial degradation [54]. The most commonly applied methods for investigating microbial plastic degradation include measuring the weight loss of polymers gravimetrically or determining polymer oxidation with Fourier-transform infrared spectroscopy [55]. In addition, scanning electron microscopy or laser scanning confocal microscopy has been applied to visualize ongoing plastic fragmentation and biofilm formation on the plastic surface [56, 57]. These approaches can typically not distinguish between abiotic and biotic degradation pathways, are not sensitive, and/or require time-consuming experimental procedures. Furthermore, none of these methods are suitable to directly trace carbon from the polymer into degradation products or microbial biomass (which would provide unambiguous proof for biodegradation) and they are also not suitable for determining microbial degradation kinetics.

Here, we show that the fungus *Rhodotorula mucilaginosa*, which we isolated from plastic debris from a North Sea laboratory microcosm, is capable of degrading UV-treated polyethylene. We incubated this fungus with virgin (-UV) and UV-irradiated ^13^C-labeled polyethylene and traced the polyethylene-derived carbon from the polymer source to the terminal oxidation product CO_2_ and measured polyethylene mineralization rates with unprecedented sensitivity. Furthermore, we visualized the assimilation of plastic-derived ^13^C into single cells of *R. mucilaginosa* by nanometer scale secondary ion mass spectrometry (nanoSIMS). This approach allows following the fate of plastic-derived carbon in marine ecosystems.

## Materials and methods

### Assays with ^13^C-polyethylene and *Rhodotorula mucilaginosa*

Details of the experimental setups are provided in the Supplementary appendix. In brief, *Rhodotorula mucilaginosa* (Supplementary Table S1) was isolated from a 350 L laboratory seawater microcosm containing a variety of plastic items mostly retrieved from the North Sea [58]. Two separate assays were performed to test the ability of the cultured *R. mucilaginosa* cells to mineralize polyethylene and assimilate polyethylene-derived carbon. The first approach involved incubating *R. mucilaginosa* cells in sealed bottles containing ~1–2 mg of ^13^C-labeled polyethylene (≥99% ^13^C, Sigma-Aldrich), either UV-treated or untreated, and measuring the transfer of ^13^C-label into the CO_2_ pool and cellular biomass (<1 week of incubation). The first assay was performed using ^13^C-labeled polyethylene as the sole energy and organic carbon source. In this experiment, the cultured cells were washed twice with sterile and autoclaved seawater, centrifuged (4000×g) at room temperature for 5 min, and “starved” for a week in sterile seawater at 25 °C to allow consumption of potentially remaining sucrose from medium prior to the incubation with polyethylene. In the second assay, we tested the ability of *R. mucilaginosa* to utilize polyethylene in the presence of another, more labile carbon source. In this setup, in addition to ^13^C-labeled polyethylene, the incubations also contained sucrose-based culture medium (10% of total liquid volume, 90% of seawater). Both assays included three treatments, each performed in triplicates: (i) ^13^C-labeled polyethylene and *R. mucilaginosa*, (ii) UV-treated ^13^C-labeled polyethylene and *R. mucilaginosa*, and (iii) uninoculated control with UV-treated ^13^C-labeled polyethylene.

### Quantification of polyethylene degradation rates

The rate of polyethylene degradation in assays with polyethylene as the sole carbon source was determined from the increase in the total amount of ^13^C-CO_2_ in the incubation bottles over time. Firstly, the identity and concentration of the headspace CO_2_ gas were measured using gas chromatography with quadrupole mass spectrometry and flame ionization detection, respectively. The isotopic composition of headspace CO_2_ was then determined by isotope ratio mass spectrometry (GC-IRMS; details in the Supplementary Material). The pH of the liquid phase was determined with a pH meter (Mettler-Toledo Seven compact S210) at the end of the incubation. Concentrations of dissolved inorganic carbon (DIC) in the liquid phase were then determined from headspace CO_2_ and pH measurements [59, 60]. CO_2_ and DIC concentrations were then converted to the total amount of CO_2_ per incubation bottle (∑CO_2_). Excess ^13^C in the headspace CO_2_ and DIC (liquid phase) pool was calculated from the change in δ^13^C-CO_2_, which is equivalent to a change in the fractional abundance of ^13^C (^13^F [61]).

1 $${\,}^{13}F_{{{{{{\rm{sample}}}}}}} = \frac{{\left[ {\left( {\frac{{{\,}^{13}{{{{{\rm{C}}}}}}}}{{{\,}^{12}{{{{{\rm{C}}}}}}}}} \right)_{{{{{{{{\rm{standard}}}}}}}}} \times \left( {\frac{{\delta {\,}^{13}{{{{{\rm{C}}}}}} - {{{{{\rm{CO}}}}}}_2}}{{1000}} + 1} \right)} \right]}}{{1 + \left\lfloor {\left( {\frac{{{\,}^{13}{{{{{\rm{C}}}}}}}}{{{\,}^{12}{{{{{\rm{C}}}}}}}}} \right)_{{{{{{{{\rm{standard}}}}}}}}} \times \left( {\frac{{\delta {\,}^{13}{{{{{\rm{C}}}}}} - {{{{{\rm{CO}}}}}}_2}}{{1000}} + 1} \right)} \right\rfloor }}$$

Here, δ^13^C-CO_2_ is the measured stable isotope composition of headspace CO_2,_ and ^13^C/^12^C_standard_ is the stable carbon isotope ratio of the Vienna PeeDee Belemnite standard (VPDB). The only ^13^C-organic carbon source in our incubations was polyethylene. Hence, an increase in ^13^F in the ∑CO_2_ pool is caused by an excess amount of ^13^C (^13^C_ex_) originating from the added substrate:

2 $${\,}^{13}{{{{C}}}}_{ex }= ({\,}^{13}F_{t_{n}} -{\,}^{13}F_{t_{o}}) \times \sum {{{{{\rm{CO}}}}}}_2$$

**Fig. 1:**
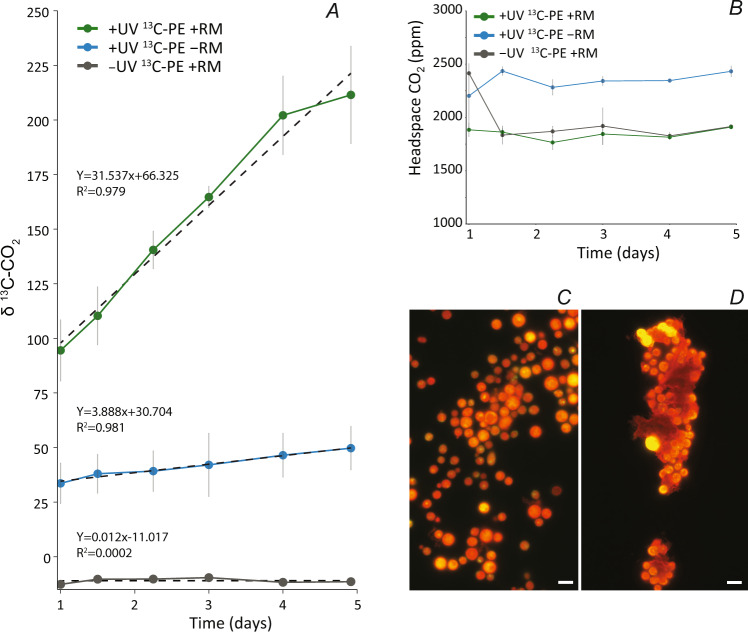
Results of polyethylene degradation assays with *R. mucilaginosa*. **A** Development of δ^13^C-CO_2_ values in incubations with with ^13^C-polyethylene (PE) as the sole carbon source with prior UV-treatment (+UV) and without (-UV) with *R. mucilaginosa* (RM) and with ^13^C-polyethylene (PE) with prior UV-treatment without *R. mucilaginosa*. The strong increase in δ^13^C-CO_2_ in incubations with *R. mucilaginosa* provides evidence for the mineralization of polyethylene-derived ^13^C. **B** CO_2_ in the headspace (15 mL) of the incubations. All data are shown as averages ± standard deviation (*n* = 3). **C**, **D** Microscopic images of *R. mucilaginosa* stained with acridine orange and visualized with ×100 magnification, **C** *R. mucilaginosa* cells grown in sucrose-based medium (control), **D** *R. mucilaginosa* cells of incubation assay with untreated polyethylene as sole carbon source. Cells are attached to the polyethylene particles and form densely packed aggregates with polyethylene particles. The scale bar is equal to 5 µm.

The change over time in ^13^C_ex_ (Δ^13^C_ex_) was calculated from the change in δ^13^C (thus ^13^F), i.e., the slope of δ^13^C (Fig. 1A) and ∑CO_2_ at the endpoint of the experiment. Δ^13^C_ex_ is proportional to the mineralization rate of polyethylene-derived carbon. Mineralization rates were expressed as %-degradation of the initially added ^13^C-polyethylene (% d^–1^,% yr^–1^ Supplementary Table 2).

### Quantification of polyethylene-derived carbon assimilation

Small aliquots (150 µL) of the liquid with the *R. mucilaginosa* biomass collected at the end of incubation experiments were filtered onto polycarbonate filters (0.2 µm pore size, Millipore), washed three times with 1×PBS, and placed in a desiccator at room temperature to dry. Chemical fixation was not performed, thus avoiding dilution of the isotope label. These samples were subsequently measured with nanoSIMS to quantify the ^13^C labeling of individual *R. mucilaginosa* cells (details in the Supplementary Material). In this analysis, *R. mucilaginosa* cells from the inoculum were used as controls. NanoSIMS data were processed and analyzed using Look@NanoSIMS [62].

## Results

### Polyethylene degradation rates

We conducted activity assays with ^13^C-labeled polyethylene as the sole organic carbon and energy source to investigate the potential of *R. mucilaginosa* to degrade and mineralize polyethylene. We used either untreated ^13^C-polyethylene or ^13^C-polyethylene that was irradiated prior to incubation with a UV A/B dose corresponding to ~50 and ~125 days of UV irradiation at the sea surface in the subtropical and temperate regions, respectively [63].

We found that the δ^13^C-values in the headspace CO_2_ pool increased linearly by 117‰ over 5 days of incubation when the experiment was conducted with *R. mucilaginosa* and UV-treated ^13^C-polyethylene (Fig. 1A). In contrast, during the same time interval, the δ^13^C-CO_2_ values increased by 16‰ in the experiments where the UV-treated ^13^C-polyethylene was incubated without *R. mucilaginosa*. The δ^13^C-CO_2_ values did not increase in incubations with untreated ^13^C-polyethylene and *R. mucilaginosa*. The CO_2_ concentrations in the headspace remained relatively constant, ~1900 ppm in incubations with fungal inoculum and ~2400 ppm in incubations without fungi (Fig. 1B). With respect to the headspace volumes, this amounts to a total of ~1.2 and 1.5 µmol CO_2_, respectively. Plastic mineralization was determined from the total amount of CO_2_ per incubation bottle (∑CO_2_), comprising CO_2_ in the headspace and DIC in the liquid phase. This, combined with the change in δ ^13^C-CO_2_, translates to an excess production of ^13^C in the carbonate system of the incubations (^13^F, see Materials and methods). The ^13^C excess production is equivalent to the mineralization rate of polyethylene-derived carbon. Based on linear regression analysis, the increase in δ^13^C in incubations with UV-treated ^13^C-polyethylene and with *R. mucilaginosa* was 31.5‰ d^–1^ (Fig. 1A). This is equivalent to an absolute change in ^13^F (Δ^13^F) of 0.000345 d^–1^ (Supplementary Table S2). Together with an average ∑CO_2_ of 57.7 µmol, this translates to a Δ^13^C_ex_ of 0.0199 µmol d^–1^. In control incubations with UV-treated ^13^C-polyethylene but without *R. mucilaginosa* the increase in δ^13^C of 3.9‰ d^–1^ translates to a Δ^13^F of 0.000043 d^–1^ and together with a ∑CO_2_ value of 151.7 µmol to a Δ^13^C_ex_ of 0.0064 µmol d^–1^. We attribute this excess production to ongoing radical chain reactions leading to polymer oxidation and CO_2_ production even after UV exposure has stopped [3, 24]. In contrast, δ^13^C increased insubstantially by only 0.01‰ d^–1^ in incubations with *R. mucilaginosa* but where the polyethylene was not irradiated with UV light prior to incubation. In these incubations, the Δ^13^F was 0.0000001 d^–1^, ∑CO_2_ was 48.2 µmol and Δ^13^C_ex_ was hence 0.000006 µmol d^–1^. For the incubations with UV-treated polyethylene, we further calculated net polyethylene mineralization rates. Considering the ~1.8 and ~1.7 mg ^13^C-polyethylene added to incubations with and without fungi, the Δ^13^C_ex_ in these incubations translates to polyethylene mineralization rates of 0.016% d^–1^ and 0.006% d^–1^, respectively. Hence, the net *R. mucilaginosa* mediated polyethylene degradation amounts to 0.01% d^–1^, or 3.8% yr^–1^. The *R. mucilaginosa* cell counts in this incubation were 3.32 × 10^5^ ± 1.27 × 10^4^ ml^–1^ resulting in mineralization rates of 6.05 × 10^–5^ nmol polyethylene cell^–1^ d^–1^ or 0.022 nmol polyethylene cell^–1^ yr^–1^.

### nanoSIMS

**Fig. 2:**
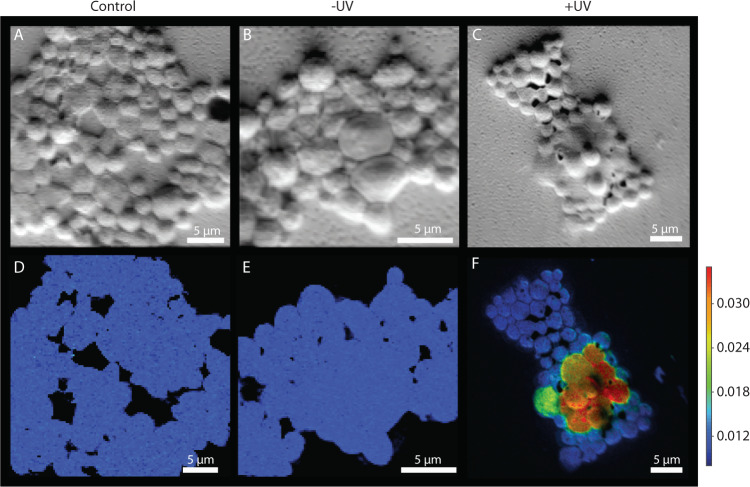
NanoSIMS images of *R. mucilaginosa* cells. **A** Secondary electron image of *R. mucilaginosa* cells in the medium (Control), **B** *R. mucilaginosa* cells with untreated ^13^C-polyethylene (-UV), **C** *R. mucilaginosa* cells with UV-treated ^13^C-polyethylene (+UV). The respective ^13^F-values of *R. mucilaginosa* cells are presented on panels **D**–**F**. Highest incorporation of ^13^C label was found in *R. mucilaginosa* cells with UV-treated ^13^C-polyethylene as depicted by warm colors in panel **F**.

**Fig. 3:**
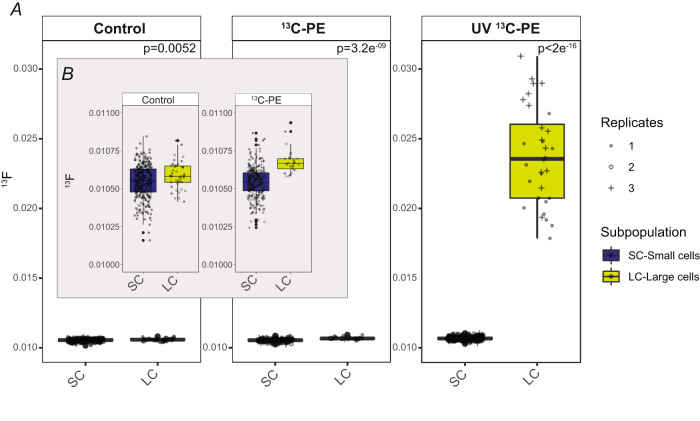
Box and whiskers plot of ^13^F-values of small (SC) and larger (LC) *R. mucilaginosa* cells. **A** Cells in medium (Control), with untreated ^13^C-polyethylene (^13^C-PE), UV-treated polyethylene (UV ^13^C-PE). **B** A zoom in of Control and ^13^C-polyethylene treatments. Differences in ^13^F-values were statistically significant for ^13^C-polyethylene and UV ^13^C-polyethylene for small and enlarged cells. *p* values (*t*-test) are indicated in the plot. Boxplots depict the median, the first and third quartiles, the upper/lower whiskers that extend from the hinge to the largest/smallest value no further than 1.5× of the interquartile range from the hinge. Outliers are represented as big dots.

In all sample sets, *R. mucilaginosa* cells featured similar cellular properties with two distinct subpopulations: enlarged cells of ~4 µm (likely growing/dividing cells or polyploids), and smaller cells with a diameter of ~2 µm (Fig. 2). Fluorescence microscopy showed a homogenous cell suspension (without formation of cell aggregates) of *R. mucilaginosa* cells in the original inoculum (Fig. 1C). In contrast, *R. mucilaginosa* cells adhered to plastic particles and clumped together in the incubations with added polyethylene (Fig. 1D). For nanoSIMS measurements of *R. mucilaginosa* cells, we analyzed three sample sets: (i) the original inoculum, (ii) after incubation with ^13^C-labeled polyethylene without and (iii) with UV-treatment. We measured a total of 1144 regions of interest (ROIs, i.e., corresponding to 1144 individual cells; Table 1). In control incubations with no added polyethylene, both cell types had mean ^13^F-values of 0.0105 and 0.0106, respectively (Figs. 2, 3 and Table 1). Similarly, small cells in incubations with untreated ^13^C-polyethylene showed ^13^F-values of 0.0105, but larger cells were slightly, yet significantly ^13^C enriched with ^13^F-values of 0.0107 (*p* < 0.001, Dunn’s Kruskal–Wallis test with Bonferroni correction). The small cells in incubations with UV-treated ^13^C-polyethylene had similar ^13^C-enrichment with ^13^F-values of 0.0107. However, the most substantial ^13^C-enrichment with ^13^F-values of 0.024 was found in large *R. mucilaginosa* cells with UV-treated ^13^C-polyethylene. This was significantly higher than any other cell group, irrespective of the added substrate (*p* < 0.001, Dunn’s Kruskal–Wallis test with Bonferroni correction). Results of the statistical analysis are presented in Supplementary Table S3.

**Table 1:** ^13^F-values of cells in the medium (Control), *R. mucilaginosa* cells with untreated ^13^C-polyethylene (^13^C-PE) and UV-treated ^13^C-polyethylene (UV ^13^C-PE).

Treatment	Cell type	*n*	Mean ^13^F	SD
Control	Small	305	0.0105	0.0001
Control	Large	41	0.0106	0.0001
^13^C-PE	Small	272	0.0105	0.0001
^13^C-PE	Large	26	0.0107	0.0001
UV ^13^C-PE	Small	467	0.0107	0.0001
UV ^13^C-PE	Large	33	0.0240	0.0037

Values are presented per subpopulation (small or large *R. mucilaginosa* cells) with the number (n) of cells/region of interest (ROIs) determined. SD is the calculated standard deviation.

To investigate if *R. mucilaginosa* also metabolizes polyethylene-derived carbon in the presence of other, potentially more accessible carbon sources, we additionally incubated *R. mucilaginosa* in seawater containing 10% of MS medium, with and without UV-treated ^13^C-polyethylene. NanoSIMS analysis revealed slightly ^13^C-enriched cells (^13^F-values of ~0.012) in incubations where UV-treated ^13^C-labeled polyethylene was added (Supplementary Fig. S1). In these incubations, the ^13^C-enrichment was more homogenous over all cell types. However, the ^13^C-enrichment was much lower compared to the incubations where *R. mucilaginosa* cells where exposed to UV-treated polyethylene as the sole carbon source and where large cells showed ^13^F-values of 0.024 (see above). No incorporation of the ^13^C to *R. mucilaginosa* cells was recorded in incubations where the ^13^C-polyethylene was not UV-treated.

## Discussion

Microbial degradation, in conjunction with physicochemical processes, may be an important pathway in breaking down plastic litter in the marine environment. Yet, little is known about potential microorganisms degrading marine plastic litter because most quantitative techniques do not allow resolving degradation in the sub-percent range, nor do the experimental setups provide unambiguous proof of microbial utilization of plastics. In this work, we present data from short (<1 week) experimental assays that allowed us to quantitatively measure plastic mineralization rates. In addition, we traced and visualized the assimilation of plastic-derived carbon into individual cells of *R. mucilaginosa*. Central to this approach is the utilization of isotopically labeled polyethylene, containing a high degree of ^13^C instead of ^12^C, and tracing the polyethylene-derived ^13^C in the degradation product CO_2_ and biomass.

### Degradation of ^13^C-polyethylene

Fungal mineralization of polyethylene-derived carbon occurred in incubations with UV-treated polyethylene, indicating that initial photooxidation enhances fungal plastic degradation in the marine environment. UV-induced photooxidation leads to the formation of carbonyl and hydroxyl moieties in the polymer [24, 64]. This facilitates access for microbial enzymes and thus further plastic degradation [65]. Moreover, UV-induced photodegradation results in the leaching of a plethora of lower molecular weight degradation products [3, 10, 23, 25, 66], which, at least to some degree, stimulate microbial activity [23, 25]. In our experiments, we did not measure photooxidation products other than CO_2_, and we can thus not determine which compounds were utilized by *R. mucilaginosa*. Nevertheless, the UV-treated ^13^C-polyethylene was washed and dried before incubation, which likely removed volatile compounds that were generated during the UV treatment. Longer chain degradation compounds and polymers with carbonyl and hydroxyl moieties [24], however, will likely have remained accessible for the fungus. For measuring degradation kinetics, we quantitatively traced ^13^C from isotopically labeled ^13^C-polyethylene into the terminal oxidation product CO_2_. Mineralization rates of UV-treated polyethylene with *R. mucilaginosa* amounted to 0.01% d^–^^1^; extrapolated, this translates to degradation rates of 3.8% yr^–1^ of the initially added polyethylene. These microbially mediated degradation rates are difficult to compare with previously reported rates from literature. Polymer weight loss over time is a frequently used parameter for measuring plastic degradation rates [67–69]. Polymer mass may, however, be lost as a result of fragmentation, and during clean-up procedures, so gravimetric methods do not allow a clear-cut distinction between biotic and abiotic degradation. Furthermore, resolving environmental plastic degradation rates of a few percent per year necessitates long incubation times of months to years in order to detect gravimetric changes. Also, Fourier-transform infrared spectroscopy allowing detection of carbonyl and hydroxyl groups in the polymer backbone [68, 70] does not allow a distinction between biotic and abiotic degradation. Chemical alteration of the initial polymer matrix can be the result of microbial degradation, but also caused by physical and chemical processes such as photooxidation. In contrast, using isotopically labeled polymers offers the advantage of directly and quantitatively tracing plastic-derived carbon into the terminal degradation product CO_2_. Our methodological approach allows resolving δ^13^C-CO_2_ values of ≳1‰ with confidence, which is equivalent to a change in the ^13^C content in the ∑CO_2_ pool of ~0.001%. This detection limit is determined by the background CO_2_ level as well as the added ^13^C-substrate. For a setup resembling ours (0.1 mmol ∑CO_2_, 1 mg of 99 atom % ^13^C-labeled polyethylene), degradation of ~0.002% of the added polyethylene is detectable. This method is consequently orders of magnitude more sensitive than more commonly used methods and also allows measuring plastic degradation rates in much shorter time periods of days to weeks as opposed to months to years.

### Biomass incorporation of polyethylene-derived carbon

We measured stable carbon isotope ratios of single fungal cells using nanoSIMS, a technique that, in scanning mode, allows collecting images of isotopic composition with a spatial resolution in the nanometer scale [71]. Previously conducted incubations with the ^13^C-labeled biodegradable/compostable plastic poly (butylene adipate-co-terephthalate) (PBAT) and agricultural soil demonstrated incorporation of ^13^C from the PBAT into fungal hyphae and other unicellular organisms [72]. Also, labeled ^13^C-polyethylene has been used to trace plastic-derived carbon in food webs of a boreal-lake and artificial humic waters [73]. Our findings of highly ^13^C enriched cells provide, for the first time, unambiguous proof of the assimilation of polyethylene-derived carbon by a marine-derived fungus. The nanoSIMS measurements show that the incorporation of polyethylene-derived ^13^C was not homogenous into all fungal cells. We detected two subpopulations of cells, one with significant but low ^13^C-enrichment and a second population with an extraordinary high enrichment. The cells with higher enrichment were larger in size, thus probably growing or polyploids. Potentially, these cells were in closer proximity to the plastic substrate and incorporated more of the ^13^C label. The vicinity of the fungal cells to the plastic particles is likely crucial in the degradation process as the plastic substrate is not a soluble compound and is thus not homogenously distributed on a µm scale. Similar to kinetic measurements, we found higher assimilation of polyethylene-derived carbon in *R. mucilaginosa* cells in incubations with UV-treated polyethylene. Moreover, using nanoSIMS, we could also find a ^13^C-enrichment in *R. mucilaginosa* cells in incubation with ^13^C-polyethylene that was not UV irradiated prior to the incubation experiments. This indicates that the fungus can also degrade the virgin (-UV) polymer, though to a much lesser degree, as shown by the lower biomass ^13^C-enrichment when compared with our measurements with UV-treated polyethylene. Nevertheless, this will need further confirmation in future studies addressing molecular weight distribution of the polymer and the degree to which it contains short-chain impurities.

Finally, in incubations containing a sucrose-based medium and UV-treated ^13^C-polyethylene as potential carbon substrates, we observed label incorporation into *R. mucilaginosa* cells, although to a much lesser degree. However, this shows that *R. mucilaginosa* utilizes polyethylene-derived carbon also in the presence of more bioavailable carbon substrates that are easier to metabolize. This consequently implies that *R. mucilaginosa* could also metabolize polyethylene-derived carbon in the marine environment, where plastic only makes up a fraction of the plethora of available organic matter compounds.

### Environmental implications

The role and identity of plastic-degrading microbes in the marine environment, specifically fungi is unconstrained. Nevertheless, many different fungi colonize plastic marine debris [49, 51, 74, 75] and thus potentially have access to plastic as a carbon source. Though several plastic-degrading fungi have been identified and isolated from terrestrial and freshwater environments [76–78], only two marine fungi belonging to the Ascomycota: *Zalerion maritimum* and *Alternaria alternata* are known to degrade plastic [52, 53]. Our isolated fungus, *R. mucilaginosa*, is a marine yeast belonging to the Basidiomycota. Yeasts have generally not been described as plastic degraders; however, yeasts are generally ubiquitous throughout fresh, marine, and deep sea environments [79]. Reported cell counts range from 10 to 50 cells L^–1^ in seawater compared to up to 500 cells L^–1^ in rivers [80]. The genus *Rhodotorula* is widespread throughout all ecosystems [81], including aquatic environments. *R. mucilaginosa* has been detected in lakes [82], hypersaline inland seas [83], arctic glaciers [84], and the deep sea [85] and was found as a dominant fungus on marine plastics [51]. *R. mucilaginosa* is common in bioremediation practices, where *R. mucilaginosa* strains have shown potential as nitrobenzene [86] and acrylamide degraders [87]. Similarly, *R. mucilaginosa* has been tested for its ability to remove phenolic compounds from olive mill wastewater [88, 89]. *R. mucilaginosa* thus seems to be able to break down a variety of hydrocarbon/hydrocarbon-like compounds. Together with our findings, this consequently suggests that *Rhodotorula mucilaginosa* is a potentially important plastic degrader in a wide range of marine environments. Furthermore, as this yeast has been found in a diversity of aquatic systems globally, it may hence degrade plastics there too.

## Conclusion

Fungi in the marine environment are highly understudied despite their prevalence in the ocean. With the aid of stable isotope assays, we provide unambiguous proof that the fungus *Rhodotorula mucilaginosa* uses polyethylene-derived carbon for cellular incorporation and energy gain. The ability of *R. mucilaginosa* to utilize plastic-derived carbon in the presence of other, high-energy-yielding carbon substrates also indicates that fungal plastic degradation can indeed proceed in the natural environment. Our results confirm that initial plastic photooxidation is a key process in making plastic available for subsequent microbial degradation. Most produced and discarded plastic types such as polyethylene and polypropylene float at the ocean surface and will consequently be subjected to photooxidation so that fungal degradation can commence there. At least parts of the vast amounts of plastic litter in the ocean may thus serve as a carbon source for fungi and possibly other microbes, too.

## Supplementary information

Supplementary material

Figure S1

Table S1

Table S2

Table S3

## Data Availability

The authors declare that the data supporting the findings of this study are available within the paper and the raw data have been deposited to 10.25850/nioz/7b.b.gf. The isolated *R. mucilaginosa* strain was deposited to the culture collection of fungi and yeasts at Westerdijk Institute with identifier ID11602.

## References

[1] Geyer R, Jambeck JR, Law KL (2017). Production, use, and fate of all plastics ever made. Sci Adv.

[2] Jambeck JR, Geyer R, Wilcox C, Siegler TR, Perryman M, Andradyet A (2015). Plastic waste inputs from land into the ocean. Science.

[3] Ward CP, Armstrong CJ, Walsh AN, Jackson JH, Reddy CM (2019). Sunlight converts polystyrene to carbon dioxide and dissolved organic carbon. Environ Sci Technol Lett.

[4] Ostle C, Thompson RC, Broughton D, Gregory L, Wootton M, Johns DG (2019). The rise in ocean plastics evidenced from a 60-year time series. Nat Commun.

[5] Lebreton L, Slat B, Ferrari F, Sainte-Rose B, Aitken J, Marthouseet R (2018). Evidence that the Great Pacific Garbage Patch is rapidly accumulating plastic. Sci Rep.

[6] MacLeod M, Arp HPH, Tekman MB, Jahnke A (2021). The global threat from plastic pollution. Science.

[7] Lebreton LCM, van der Zwet J, Damsteeg J-W, Slat B, Andrady A, Reisser J (2017). River plastic emissions to the world’s oceans. Nat Commun.

[8] Law KL (2017). Plastics in the marine environment. Ann Rev Mar Sci.

[9] Weiss L, Ludwig W, Heussner S, Canals M, Ghiglione J-F, Estournelet C (2021). The missing ocean plastic sink: gone with the rivers. Science.

[10] Andrady AL (2011). Microplastics in the marine environment. Mari Pollut Bull.

[11] Liss PS. Microplastics: all up in the air? In: EGU General Assembly Conference Abstracts. 2020.

[12] Onink V, Jongedijk CE, Hoffman MJ, van Sebille E, Laufkötter C (2021). Global simulations of marine plastic transport show plastic trapping in coastal zones. Environ Res. Lett.

[13] Eriksen M, Lebreton LCM, Carson HS, Thiel M, Moore CJ, Borerroet JC (2014). Plastic pollution in the world’s oceans: more than 5 trillion plastic pieces weighing over 250,000 tons afloat at sea. PLOS ONE.

[14] van Sebille E, Wilcox C, Lebreton L, Maximenko N, Hardesty BD, van Franekeret JA (2015). A global inventory of small floating plastic debris. Environ Res. Lett.

[15] Choy CA, Robison BH, Gagne TO, Erwin B, Firl E, Haldenet RU (2019). The vertical distribution and biological transport of marine microplastics across the epipelagic and mesopelagic water column. Sci Rep.

[16] Pabortsava K, Lampitt RS (2020). High concentrations of plastic hidden beneath the surface of the Atlantic Ocean. Nat Commun.

[17] Egger M, Sulu-Gambari F, Lebreton L (2020). First evidence of plastic fallout from the North Pacific Garbage Patch. Sci Rep.

[18] Kane IA, Clare MA, Miramontes E, Wogelius R, Rothwell JJ, Garreauet P (2020). Seafloor microplastic hotspots controlled by deep-sea circulation. Science.

[19] Krause S, Molari M, Gorb EV, Gorb SN, Kossel E, Haeckel M (2020). Persistence of plastic debris and its colonization by bacterial communities after two decades on the abyssal seafloor. Sci Rep.

[20] Cozar A, Echevarria F, Gonzalez-Gordillo JI, Irigoien X, Ubeda B, Hernandez-Leonet S (2014). Plastic debris in the open ocean. Proc Natl Acad Sci USA.

[21] Wayman C, Niemann H (2021). The fate of plastic in the ocean environment – a mini review. Environ Sci Proces Impacts.

[22] Roy PK, Titus S, Surekha P, Tulsi E, Deshmukh C, Rajagopal C (2008). Degradation of abiotically aged LDPE films containing pro-oxidant by bacterial consortium. Polym Degrad Stab.

[23] Romera-Castillo C, Pinto M, Langer TM, Álvarez-Salgado XA, Herndl GJ (2018). Dissolved organic carbon leaching from plastics stimulates microbial activity in the ocean. Nat Commun.

[24] Gewert B, Plassmann M, Sandblom O, MacLeod M (2018). Identification of chain scission products released to water by plastic exposed to ultraviolet light. Environ Sci Technol Lett.

[25] Zhu L, Zhao S, Bittar TB, Stubbins A, Li D (2020). Photochemical dissolution of buoyant microplastics to dissolved organic carbon: rates and microbial impacts. J Hazard Mater.

[26] Tanasupawat S, Takehana T, Yoshida S, Hiraga K, Oda K (2016). *Ideonella sakaiensis* sp. nov., isolated from a microbial consortium that degrades poly(ethylene terephthalate). Int J Syst Evol Microbiol.

[27] Palm GJ, Reisky L, Böttcher D, Müller H, Michels EAP, Walczaket MC (2019). Structure of the plastic-degrading Ideonella sakaiensis MHETase bound to a substrate. Nat Commun.

[28] Yoshida S, Hiraga K, Takehana T, Taniguchi I, Yamaji H, Maedaet Y (2016). A bacterium that degrades and assimilates poly(ethylene terephthalate). Science.

[29] Sivan A, Szanto M, Pavlov V (2006). Biofilm development of the polyethylene-degrading bacterium *Rhodococcus ruber*. Appl Microbiol Biotechnol.

[30] Gilan I, Hadar Y, Sivan A (2004). Colonization, biofilm formation and biodegradation of polyethylene by a strain of *Rhodococcus ruber*. Appl Microbiol Biotechnol.

[31] Mor R, Sivan A (2008). Biofilm formation and partial biodegradation of polystyrene by the actinomycete *Rhodococcus ruber*. Biodegradation.

[32] Sudhakar M, Doble M, Murthy PS, Venkatesan R (2008). Marine microbe-mediated biodegradation of low- and high-density polyethylenes. Int Biodeter Biodegradation.

[33] Kadri T, Cuprys A, Rouissi T, Brar SK. Microbial degradation of polyaromatic hydrocarbons. In: Bharagava RN, editor. Environmental contaminants: ecological implications and management. Singapore: Springer Singapore; 2019. 101–17.

[34] Barnes NM, Khodse VB, Lotlikar NP, Meena RM, Damare SR (2018). Bioremediation potential of hydrocarbon-utilizing fungi from select marine niches of India. 3 Biotech.

[35] Harms H, Schlosser D, Wick LY (2011). Untapped potential: exploiting fungi in bioremediation of hazardous chemicals. Nat Rev Microbiol.

[36] Xu J, Zhang J, Hu K, Zhang W. The relationship between lignin peroxidase and manganese peroxidase production capacities and cultivation periods of mushrooms. Microb Biotechnol. 2013;6:241–7. 10.1111/j.1751-7915.2012.00365.x PMC3815919 22966760

[37] Iiyoshi Y, Tomoaki N, Yuji T (1998). Polyethylene degradation by lignin-degrading fungi and manganese peroxidase. J Wood Sci..

[38] Srikanth M, Sandeep TSRS, Sucharitha K, Godi S (2022). Biodegradation of plastic polymers by fungi: a brief review. Bioresour Bioprocess.

[39] Ameen F, Moslem M, Hadi S, Al-Sabri A (2015). Biodegradation of low density polyethylene (LDPE) by mangrove fungi from the Red Sea Coast. Prog Rubber Plast Recycl Technol.

[40] Zeghal E, Vaksmaa A, Vielfaure H, Boekhout T, Niemann H. The potential role of marine fungi in plastic degradation – a review. Front Mar Sci. 2021;8 10.3389/fmars.2021.738877.

[41] Verma N, Gupta S (2019). Assessment of LDPE degrading potential *Aspergillus* species isolated from municipal landfill sites of Agra. SN Appl Sci.

[42] Osman M, Satti SM, Luqman A, Hasan F, Shah Z, Shah AA (2018). Degradation of polyester polyurethane by *Aspergillus* sp. strain S45 isolated from soil. J Polym Environ.

[43] Munir E, Harefa RSM, Priyani N, Suryanto D (2018). Plastic degrading fungi *Trichoderma viride* and *Aspergillus nomius* isolated from local landfill soil in Medan. IOP Conf Ser Earth Environ Sci.

[44] Zhang J, Gao D, Li Q, Zhao Y, Li L, Linet H (2020). Biodegradation of polyethylene microplastic particles by the fungus *Aspergillus flavus* from the guts of wax moth *Galleria mellonella*. Sci Total Environ.

[45] Khan S, Ali SA, Ali, Thippeswamy AS (2022). Biodegradation of low density polyethylene (LDPE) by mesophilic fungus ‘*Penicillium citrinum*’ isolated from soils of plastic waste dump yard, Bhopal, India. Environ Technol.

[46] Sowmya HV, Ramalingappa M, Krishnappa, Thippeswamy B (2015). Degradation of polyethylene by Penicillium simplicissimum isolated from local dumpsite of Shivamogga district. Environ Dev Sustain.

[47] Dudek K, Cruz B, Polidoro B, Neuer S. Microbial colonization of microplastics in the Caribbean Sea. Limnol Oceanogr Lett. 2020;5:5–17.

[48] Zettler ER, Mincer TJ, Amaral-Zettler LA (2013). Life in the “plastisphere”: microbial communities on plastic marine debris. Environ Sci Technol.

[49] Kettner MT, Rojas-Jimenez K, Oberbeckmann S, Labrenz M, Grossart H-P (2017). Microplastics alter composition of fungal communities in aquatic ecosystems. Environ Microbiol.

[50] De Tender C, Devriese LI, Haegeman A, Maes S, Vangeyte J, Cattrijsseet A (2017). Temporal dynamics of bacterial and fungal colonization on plastic debris in the North Sea. Environ Sci Technol.

[51] Lacerda ALDF, Proietti MC, Secchi ER, Taylor JD (2020). Diverse groups of fungi are associated with plastics in the surface waters of the Western South Atlantic and the Antarctic Peninsula. Molecular Ecology.

[52] Paço A, Duarte K, da Costa JP, Santos PSM, Pereira R, Pereiraet ME (2017). Biodegradation of polyethylene microplastics by the marine fungus Zalerion maritimum. Sci Total Environ.

[53] Gao R, Liu R, Sun C (2022). A marine fungus *Alternaria alternata* FB1 efficiently degrades polyethylene. J Hazard Mater.

[54] Chamas A, Moon H, Zheng J, Qiu Y, Tabassum T, Janget JH (2020). Degradation rates of plastics in the environment. ACS Sustain Chem Eng.

[55] Vaksmaa A, Hernando-Morales V, Zeghal E, Niemann H. Microbial degradation of marine plastics: current state and future prospects. In: Joshi SJ, Deshmukh A, Sarma H, editors. Biotechnology for sustainable environment. Singapore: Springer Singapore; 2021. 111–54.

[56] Vaksmaa A, Knittel K, Abdala Asbun A, Goudriaan M, Ellrott A, Witteet HJ (2021). Microbial communities on plastic polymers in the Mediterranean Sea. Front Microbiol.

[57] Meides N, Mauel A, Menzel T, Altstädt V, Ruckdäschel H, Senkeret J (2022). Quantifying the fragmentation of polypropylene upon exposure to accelerated weathering. Microplast Nanoplast.

[58] Gerritse J, Leslie HA, de Tender CA, Devriese LI, Vethaak AD (2020). Fragmentation of plastic objects in a laboratory seawater microcosm. Sci Rep.

[59] Lewis E, Wallace D, Allison L. Program developed for CO_2_ system calculations. Brookhaven National Lab., Department of Applied Science, Upton, NY, 1998.

[60] Woosley RJ (2021). Evaluation of the temperature dependence of dissociation constants for the marine carbon system using pH and certified reference materials. Mar Chem.

[61] Hayes J. An introduction to isotopic calculations. US: Woods Hole Oceanographic Institution: 2004.

[62] Polerecky L, Adam B, Milucka J, Musat N, Vagner T, Kuypers MM (2012). Look@NanoSIMS–a tool for the analysis of nanoSIMS data in environmental microbiology. Environ Microbiol.

[63] Li T, Pan D, Bai Y, Li G, He X, Chenet C-TA (2015). Satellite remote sensing of ultraviolet irradiance on the ocean surface. Acta Oceanol Sin.

[64] Ainali NM, Lambropoulou D, Bikiaris D. Investigation of surface alteration of microplastics by using UV irradiation. In Proceedings. Presented at the First International Conference on “Green” Polymer Materials 2020 pp. 5–25.

[65] Awasthi S, Srivastava P, Singh P, Tiwary D, Mishra PK (2017). Biodegradation of thermally treated high-density polyethylene (HDPE) by *Klebsiella pneumoniae* CH001. 3 Biotech.

[66] Sørensen L, Groven AS, Hovsbakken IA, Del Puerto O, Krause DF, Sarnoet A (2021). UV degradation of natural and synthetic microfibers causes fragmentation and release of polymer degradation products and chemical additives. Sci Total Environ.

[67] Delacuvellerie A, Cyriaque V, Gobert S, Benali S, Wattiez R (2019). The plastisphere in marine ecosystem hosts potential specific microbial degraders including *Alcanivorax borkumensis* as a key player for the low-density polyethylene degradation. J Hazard Mater.

[68] Hou L, Xi J, Chen X, Li X, Ma W, Luet J (2019). Biodegradability and ecological impacts of polyethylene-based mulching film at agricultural environment. J Hazard Mater.

[69] Muthukumar T, Aravinthan A, Ramadoss D, Venkatesan R, Doble M (2014). Biodegradation of starch blended high density polyethylene using marine bacteria associated with biofilm formation and its isolation characterization. J Microb Biochem Technol.

[70] Cai L, Wang J, Peng J, Wu Z, Tan X (2018). Observation of the degradation of three types of plastic pellets exposed to UV irradiation in three different environments. Sci Total Environ.

[71] Nuñez J, Renslow R, CliffIII JB, Anderton CR (2018). NanoSIMS for biological applications: current practices and analyses. Biointerphases.

[72] Zumstein MT, Schintlmeister A, Nelson TF, Baumgartner R, Woebken D, Wagneret M (2018). Biodegradation of synthetic polymers in soils: tracking carbon into CO(2) and microbial biomass. Sci Adv.

[73] Taipale SJ, Peltomaa E, Kukkonen JVK, Kainz MJ, Kautonen P, Tiirola M (2019). Tracing the fate of microplastic carbon in the aquatic food web by compound-specific isotope analysis. Sci Rep.

[74] Kettner MT, Oberbeckmann S, Labrenz M, Grossart H-P (2019). The eukaryotic life on microplastics in Brackish ecosystems. Front Microbiol.

[75] Oberbeckmann S, Osborn AM, Duhaime MB (2016). Microbes on a bottle: substrate, season and geography influence community composition of microbes colonizing marine plastic debris. PLOS ONE.

[76] Yamada-Onodera K, Mukumoto H, Katsuyaya Y, Saiganji A, Tani Y (2001). Degradation of polyethylene by a fungus, *Penicillium simplicissimum* YK. Polym Degrad Stab.

[77] Brunner I, Fischer M, Rüthi J, Stierli B, Frey B (2018). Ability of fungi isolated from plastic debris floating in the shoreline of a lake to degrade plastics. PLOS ONE.

[78] Ojha N, Pradhan N, Singh S, Barla A, Shrivastava A, Khatuaet P (2017). Evaluation of HDPE and LDPE degradation by fungus, implemented by statistical optimization. Sci Rep.

[79] Nagahama T. Yeast biodiversity in freshwater, marine and deep-sea environments. In: Péter G, Rosa C, editors. Biodiversity and ecophysiology of yeasts. Berlin, Heidelberg: Springer Berlin Heidelberg; 2006. 241–62.

[80] Hagler A, Hagler L (1978). The yeasts of fresh water and sewage. An Microbiol (Rio J).

[81] Kurtzman CP, Fell JW, Boekhout T. The yeasts: a taxonomic study. Elsevier: Amsterdam, the Netherlands: 2011.

[82] Brandão LR, Libkind D, Vaz ABM, Espírito Santo LC, Moliné M, de Garcíaet V (2011). Yeasts from an oligotrophic lake in Patagonia (Argentina): diversity, distribution and synthesis of photoprotective compounds and extracellular enzymes. FEMS Microbiol Ecol.

[83] Fotedar R, Kolecka A, Boekhout T, Fell JW, Al-Malki A, Zeyaraet A (2018). Fungal diversity of the hypersaline Inland Sea in Qatar. Botanica Marina.

[84] Butinar L, Spencer-Martins I, Gunde-Cimerman N (2007). Yeasts in high Arctic glaciers: the discovery of a new habitat for eukaryotic microorganisms. Antonie Van Leeuwenhoek.

[85] Richards TA, Jones MDM, Leonard G, Bass D (2012). Marine fungi: their ecology and molecular diversity. Ann Rev Mar Sci.

[86] Zheng C, Zhou J, Wang J, Qu B, Wang J, Luet H (2009). Aerobic degradation of nitrobenzene by immobilization of Rhodotorula mucilaginosa in polyurethane foam. J Hazard Mater.

[87] Rahim MBH, Syed MA, Shukor MY (2012). Isolation and characterization of an acrylamide-degrading yeast *Rhodotorula* sp. strain MBH23 KCTC 11960BP. J Basic Microbiol.

[88] Jarboui R, Magdich S, Ayadi RJ, Gargouri A, Gharsallah N, Ammar E (2013). Aspergillus niger P6 and *Rhodotorula mucilaginosa* CH4 used for olive mill wastewater (OMW) biological treatment in single pure and successive cultures. Environ Technol.

[89] Jarboui R, Baati H, Fetoui F, Gargouri A, Gharsallah N, Ammar E (2012). Yeast performance in wastewater treatment: case study of *Rhodotorula mucilaginosa*. Environ Technol.

