# Supplementary material for: Polyethylene degradation and assimilation by the marine yeast Rhodotorula mucilaginosa
Source: ISME Commun. 2023 Jul 10;3:68. doi: 10.1038/s43705-023-00267-z (PMC10330194; doi:10.1038/s43705-023-00267-z)
Supplement: Supplementary file 1 — Supplementary material [file 43705_2023_267_MOESM1_ESM.pdf]

Supplementary Information for

Polyethylene degradation and utilization by the marine yeast *Rhodotorula mucilaginosa*

Annika Vaksmaa[1], Lubos Polerecky[2], Nina Dombrowski[1], Michiel V.M Kienhuis[2], Ilsa Posthuma[1], Jan Gerritse[3], Teun Boekhout [4,5], Helge Niemann[1,2]

1 NIOZ Royal Netherlands Institute for Sea Research, Department of Marine Microbiology and Biogeochemistry, the Netherlands

2 Department of Earth Sciences, Faculty of Geosciences, Utrecht University, the Netherlands

3 Deltares, Unit Subsurface and Groundwater Systems, Utrecht the Netherlands

4 Westerdijk Fungal Biodiversity Institute, the Netherlands, Institute of Biodiversity and Ecosystem Dynamics (IBED), University of Amsterdam, the Netherlands

5 College of Science, King Saud University, Saudi Arabia

*Annika Vaksmaa.

Email: annika.vaksmaa@nioz.nl

Supplementary Information Text

Isolation and culturing of *Rhodotorula mucilaginosa*

Rhodotorula mucilaginosa was isolated from an open 350 l microcosm, which had been inoculated with seawater from the North Sea and plastic fragments and objects, and was maintained at a 12 h light and 12 h dark regime [1]. Isolation of *Rhodotorula mucilaginosa* was carried out by using swabs and seawater from the tank as inoculum. First these were kept in filtered (0.2 µm) seawater (Wadden Sea, Texel, Netherlands), and thereafter culturing was carried out using Murashige and Skoog (MS) medium [2]. The media was composed of 4.4 g l^{-1} of Murashige and Skoog powder (Sigma Aldrich), 1 l of autoclaved seawater, 1 mg of BAA hormone solution (1 mg ml^{-1}) (6-Benzylaminopurine, B3408, Sigma Aldrich), 0.5 mg of NAA hormone solution (1 mg ml^{-1}) (1-Naphthaleneacetic acid, N0640, Sigma Aldrich), 30 g l^{-1} of Sucrose (Sigma Aldrich), adjusted to pH 5.8. For the preparation of solid media, 8 g l^{-1} of agar (Agar Bacteriological, VWR Chemicals) was added to the MS media. The isolation was carried out first by streaking out a single colony on MS medium agar plates and once a pure culture was obtained, liquid MS medium was used.

DNA extraction, PCR amplification

One ml aliquot of the liquid culture was sampled and DNA was extracted using the Powersoil PRO DNA extraction kit (MoBio Laboratories, Inc, Carlsbad, USA). First, the aliquot was centrifuged in a 2 ml tube for 5 min at 10,000×g, the supernatant was removed, and Powerbeads were added. Then, a bead beating step of 4.55 m s^{-1} for 30 seconds 3 times with dwell time of 10 seconds was applied, replacing the original cell lysis step [3]. Further steps followed the protocol provided by the manufacturer. DNA concentrations were measured on a Qubit fluorometer by Qubit dsDNA HS Assay Kit (Invitrogen, USA) by adding 2 µl of DNA sample to 198 µl of Qubit working solution. To identify the strain, the Internal Transcribed Spacer (ITS) region was amplified by primers ITS1Fngs ('GGTCATTTAGAGGAAGTAA') and ITS4ngsUni ('CCTSCSCTTANTDATATGC') [4] and 18S rRNA gene primers NSF4/18 ('CTGGTTGATYCTGCCAGT') [5] and EukR ('TGATCCTTCTGCAGGTTCACCTAC') [6]. PCR reaction volume of 25 µl contained: 1.5 µl of each primer (10 µM) (Biolegio, the Netherlands), 5 µl of 5×HF

Phusion Buffer (ThermoFisher Scientific™), 2 µl of DNA, 2 µl of dNTP (2.5 mM, Eurogentec, Belgium), 1 µl of BSA solution (20 mg/ml, Sigma Aldrich), 0.25µl Phusion polymerase (NEB), and brought to the final volume of 25 µl with dH_2O. PCR was performed with the following thermal program: 98° C 30 sec, 35 cycles of 98° C 30 sec, 50° C 30 sec, 72° C 30 sec, followed by 72° C 7min thereafter kept at 4° C. The size and purity of the PCR product was evaluated by running a 1% agarose gel for 45min at 95V. For this, 10 µl of the PCR product was mixed with 2 µl of loading dye, prepared by mixing 1.5g of Ficoll 400 polymer (Sigma Aldrich), 0.025 g of Xylene Cyanole (CAS Sigma Aldrich) in 10 ml of PCR grade water (AccuGENE®) and loaded to the gel. For PCR product size determination, we used 5 µl of Smartladder (MW-1700-10, Eurogentec, Belgium). The PCR product was sent for Sanger sequencing at BaseClear (Leiden, Netherlands). The sequences were further processed by trimming the primers, low quality bases and making the consensus sequence (ChromasPro).

Taxonomy assignment

The identification and taxonomic verification of the isolate was carried out with ITS primers at two time points: 1. three months prior to the experiment and by using the inoculum of the activity assay, 2. prior to the experiment. Then, identification was also carried out with 18S rRNA gene primers (Table S1). The retrieved ITS sequences matched 100% (based on 540bp) to *Rhodotorula mucilaginosa* strain 2018A0762M12 (accession nr. MT378424.1). Among the top BLAST hits, identical sequences from natural environments have been retrieved from salt fields (acc nr. MT158431.1), upland forest (acc. nr MT028113.1), soil samples from waste dumping sites (acc nr. OL890694.1), and mangrove soils (acc nr. OM250076.1). The 18S rRNA gene sequences matched 100% (based on consensus sequence of 994bp) to *Rhodotorula mucilaginosa* strain TY-03 (accession nr. MT509560.1). *Rhodotorula mucilaginosa* belongs to *Sporidiobolaceae* family, *Sporidiobolales* order in the kingdom of Fungi. Our strain was deposited to the culture collection of fungi and yeasts at Westerdijk Institute with identifier ID11602.

Microscopy

The *R. mucilaginosa* culture was visualised and cells were enumerated by microscopy. Micrographs

were prepared from cells stained with acridine orange (0.016% w/v; 10.375 mg in 65 ml of a 5% formaldehyde solution) and unstained cells. These were visualized with a ZEISS AxioPhot microscope supplied with a Jenoptik ProgRes C10 plus camera. Pictures were taken with AxioVision LE Rel. 4.5 software (Fig 1C and 1D). For cell enumeration, 1 ml of the sample liquid was fixed with 25 µl of glutaraldehyde and stored at -20°C until microscopy analysis.

Activity assays with ^{13}C-labelled polyethylene

Plastic degradation was determined from the accumulation of $^{13}C-CO_2$ in incubations of *R. mucilaginosa* with ^{13}C-labelled polyethylene. In first assays prior to incubation, potential carbon sources from the MS media were removed from the fungal inoculum as these might be more competitive than plastic-carbon. This was achieved by washing the biomass twice with sterile, autoclaved seawater, centrifugation at 4000×g at room temperature (RT) for 5 min and then pre-incubating the inoculum for a week in sterile seawater at 25°C to allow consumption of potentially remaining media. ~1-2 mg of ^{13}C-polyethylene powder (≥99% ^{13}C, Sigma-Aldrich) was weighed into 25 ml quartz bottles containing 1 ml of sterile seawater. These were then irradiated with UV a/b using a Supratech HTC 400-241 (Osram, Germany) lightbulb, which emits a UV spectrum similar to solar radiation. Irradiance levels were 250W m^{-2} and the plastic samples were irradiated for ~120 hours (h). The UV dose was thus equal to ~50 days radiation at the sea surface in the subtropical Ocean or ~125 days in temperate marine regions where UV irradiance levels are ~25 W m^{-2} and ~10 W m^{-2}, respectively [7]. Thereafter, the plastic was dried to remove volatile photooxidation products, and the UV-treated plastic was re-immersed in 9 ml sterile SW. This seawater-plastic mixture was then inoculated with 1 ml of the washed *Rhodotorula mucilaginosa* culture. Similarly, we also incubated *Rhodotorula mucilaginosa* with ^{13}C-polyethylene that was not UV radiated, and we incubated UV-treated ^{13}C-polyethylene without a fungal inoculum as a control. In addition we carried out a second set of assays where the incubations with or without UV-treated ^{13}C-labelled polyethylene contained also sucrose based MS medium (10% of liquid volume). The setup of these assays was the same as in the first set, except the applied UV-treatment was 72 h. All incubations were carried out in triplicate in 25 ml quartz vials, which after a short equilibration period of ~2 h were sealed with red butyl rubber stoppers.

Analysis of the CO_2 pool

The concentration of CO_2 in the headspace of the incubation bottles with polyethylene as sole carbon source was measured every ~24 h over the total incubation time period of 118 h by gas chromatography coupled to flame ionization detection and/or mass spectrometry (GC-MS) using an Agilent 7890 GC and Agilent 5975C quadrupole MS. The identity of all gases was determined with the MS unit, which was also used to monitor the O_2 levels. CO_2 gas was reduced to methane using a methanizer and concentrations were determined by flame ionization detection. The system performance was checked daily using standard gas mixture consisting of CO_2, CO, H_2, CH_4, N_2, and O_2 in helium. The accumulation of ^{13}C in the $\sum CO_2$ pool was determined from the change of the isotopic composition ($\delta^{13}C$) of CO_2 in the headspace, which was measured using gas chromatography isotope ratio mass spectrometry (GC-IRMS). The GC-IMRS system was an Agilent 6890 GC-coupled to a Delta V Advantage IRMS (Thermo Scientific) via a Combustion III interface. Precision and reproducibility of the IRMS system was monitored with in house carbonate standards ($NaCO_2$: $\delta^{13}C$ -0.57‰ and $CaCO_2$: $\delta^{13}C$ -24.2‰), which were cross calibrated to certified standards (NBS19).

NanoSIMS analysis

At the end of the incubation, aliquots for further nanoSIMS analysis (3 times 150µl) incubation liquid was filtered in triplicate onto polycarbonate filters, washed thrice with 1×phosphate buffered saline (PBS) and placed into a desiccator unit to dry until nanoSIMS analysis. NanoSIMS measurements and data processing were caried out as previously described [8]. Briefly, just before nanoSIMS analysis, filters with dried biomass of *R. mucilaginosa* were sputter-coated with a 10 nm gold layer, cut into small circular pieces (5 mm diameter) suitable for the nanoSIMS sample holder, and imaged with a Neoscope II JCM-6000 scanning electron microscope (JEOL, Japan) to check sample quality (cell integrity and cell density). NanoSIMS measurements were performed with a NanoSIMS 50L instrument (Cameca, France). Areas of interest were first pre-sputtered with Cs^+-ions until secondary ion yields stabilized. Subsequently, the primary Cs^+-ion beam was scanned over the sample (areas between 10 µm × 10 µm and 30 µm × 30 µm in size, dwell time of 1 ms pixel^{-1}) while detecting secondary ions $^{12}C^{12}C^-$, $^{13}C^{12}C^-$, $^{16}O^-$, $^{12}C^{14}N^-$, $^{31}P^-$, and $^{32}S^-$. To increase the overall signal, the same area was imaged multiple times,

and the resulting ion count images were aligned and accumulated. NanoSIMS data were processed with the Look@NanoSIMS software [9]. Regions of interest (ROI's), corresponding to individual *R. mucilaginosa* cells, were drawn manually based on the combined images of the $^{12}C^{12}C^{-}$ and $^{12}C^{14}N^{-}$ ions. Subsequently, the cell-specific ^{13}C fractional abundance was determined from the total counts of secondary ions $^{12}C^{12}C^{-}$ and $^{13}C^{12}C^{-}$ accumulated over the ROI pixels and expressed as $^{13}F = 0.5 \times {}^{13}C^{12}C^{-}/({}^{12}C^{12}C^{-} + 0.5 \times {}^{13}C^{12}C^{-})$.

Statistical analysis

Statistical analysis of the data was performed in R version 4.1.1 [10]. Briefly, data were summarized with the ddply function of the plyr package (v1.8.6) [11] and boxplots generated with ggplot2 (v3.3.5) [12]. Groups were compared with a t-test using the stat_compare_means function of the R ggpubr package. Summary statistics were calculated using a Welch Two Sample t-test using the stats base package. A Kruskal-Wallis test was used to test for significant differences between all comparisons using the stats base package followed by a Dunn's Kruskal-Wallis test with Bonferroni correction for multiple comparisons using the dunnTest function from the FSA package (v0.9.1) [13].

References

1. Gerritse, J., et al., *Fragmentation of plastic objects in a laboratory seawater microcosm.* Scientific Reports, 2020. **10**(1): p. 10945.
2. Murashige, T. and F. Skoog, *A Revised Medium for Rapid Growth and Bio Assays with Tobacco Tissue Cultures.* Physiologia Plantarum, 1962. **15**(3): p. 473-497.
3. Vaksmaa, A., et al., *Microbial Communities on Plastic Polymers in the Mediterranean Sea.* Frontiers in Microbiology, 2021. **12**(1021).
4. Tedersoo, L., A. Tooming-Klunderud, and S. Anslan, *PacBio metabarcoding of Fungi and other eukaryotes: errors, biases and perspectives.* New Phytol, 2018. **217**(3): p. 1370-1385.
5. Hendriks, L., et al., *The Nucleotide Sequence of the Small Ribosomal Subunit RNA of the Yeast Candida albicans and the Evolutionary Position of the Fungi among the Eukaryotes.* Systematic and Applied Microbiology, 1989. **12**(3): p. 223-229.
6. Medlin, L., et al., *The characterization of enzymatically amplified eukaryotic 16S-like rRNA-coding regions.* Gene, 1988. **71**(2): p. 491-499.
7. Li, T., et al., *Satellite remote sensing of ultraviolet irradiance on the ocean surface.* Acta Oceanologica Sinica, 2015. **34**(6): p. 101-112.
8. Polerecky, L., et al., *Temporal Patterns and Intra- and Inter-Cellular Variability in Carbon and Nitrogen Assimilation by the Unicellular Cyanobacterium Cyanothece sp. ATCC 51142.* Frontiers in Microbiology, 2021. **12**.
9. Polerecky, L., et al., *Look@NanoSIMS--a tool for the analysis of nanoSIMS data in environmental microbiology.* Environ Microbiol, 2012. **14**(4): p. 1009-23.
10. RCoreTeam, *R: A language and environment for statistical computing.* R Foundation for Statistical Computing, Vienna, Austria., 2021.
11. Wickham, H., *The Split-Apply-Combine Strategy for Data Analysis.* Journal of Statistical Software, 2011. **40**(1): p. 1 - 29.
12. Wickham, H., *ggplot2: Elegant Graphics for Data Analysis*. 2016: Springer International Publishing.
13. Ogle, D., et al., *FSA: fisheries stock analysis. R package version 0.9. 1*. 2022, Vienna: R Core Team.[Google Scholar].

Figure and Table Legends Supplementary

Figure S1: NanoSIMS images of *R. mucilaginosa* cells in the incubations containing sucrose based medium (10% of liquid volume). $^{12}C^{14}N$ values (to locate the perimeter of individual fungal cells) are shown in the top row (A-C). A: *R. mucilaginosa* cells in the medium (Control) B: *R. mucilaginosa* cells with untreated ^{13}C-polyethylene (-UV), C: *R. mucilaginosa* cells with UV-treated ^{13}C-polyethylene (+UV). The respective ^{13}F values of *R. mucilaginosa* cells are presented on panels D, E and F.

Table S1. Sequences of *R. mucilaginosa.*

Table S2. Parameters determined during activity assays.

Table S3. Results of the Dunn's Kruskal-Wallis test including the values for the Z test statistic for each comparison (Z), the unadjusted p-value (P.unadj) and the Bonferroni-corrected p-values (P.adj).
