# Supplementary figures and images for: Polyethylene degradation and assimilation by the marine yeast Rhodotorula mucilaginosa
Source: ISME Commun. 2023 Jul 10;3:68. doi: 10.1038/s43705-023-00267-z (PMC10330194; doi:10.1038/s43705-023-00267-z)

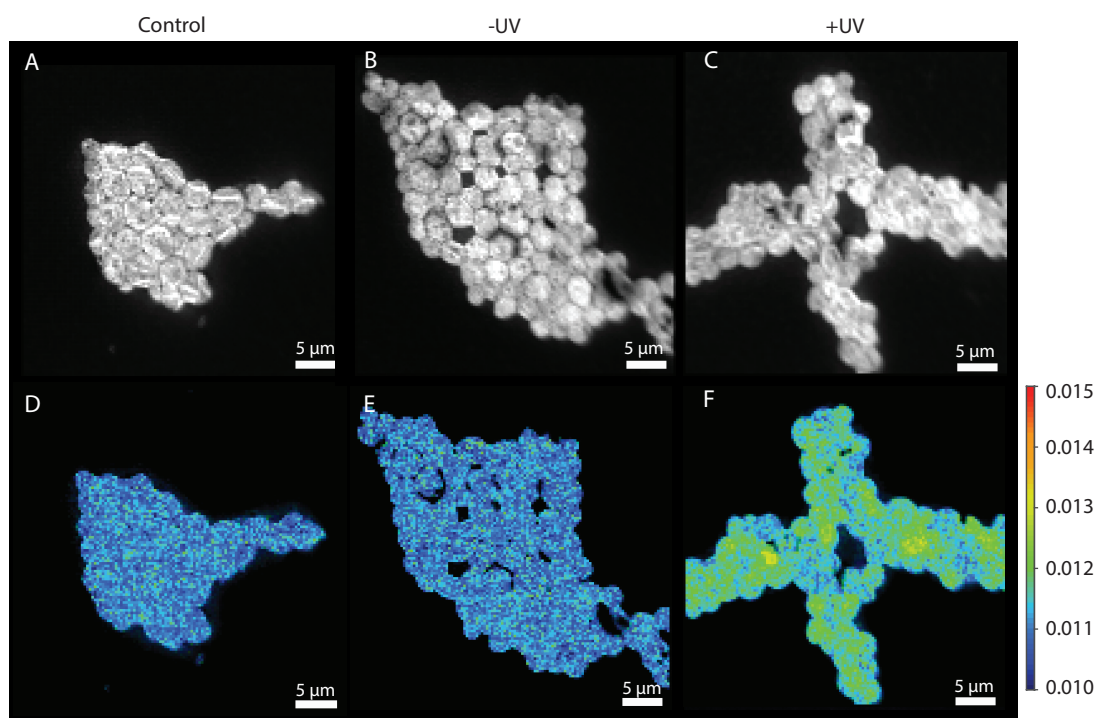

Supplement: Supplementary file 2 — Figure S1 [file 43705_2023_267_MOESM2_ESM.pdf]
