# Supplementary material for: Polyethylene degradation and assimilation by the marine yeast Rhodotorula mucilaginosa
Source: ISME Commun. 2023 Jul 10;3:68. doi: 10.1038/s43705-023-00267-z (PMC10330194; doi:10.1038/s43705-023-00267-z)
Supplement: Supplementary file 3 — Table S1 [file 43705_2023_267_MOESM3_ESM.docx]

Table S1. Taxonomy assignment, sequences of *R. mucilaginosa*

|  | Nucleotide sequence (3’-5’) |
| --- | --- |
| Sequence of the isolate  (553 nucleotides)  ITS1Fngs and ITS4ngsUni | >CTGCGGAAGGATCATTAGTGAATATAGGACGTCCAACTTAACTTGGAGTCCGAACTCTCACTTTCTAACCCTGTGCACTTGTTTGGGATAGTAACTCTCGCAAGAGAGCGAACTCCTATTCACTTATAAACACAAAGTCTATGAATGTATTAAATTTTATAACAAAATAAAACTTTCAACAACGGATCTCTTGGCTCTCGCATCGATGAAGAACGCAGCGAAATGCGATAAGTAATGTGAATTGCAGAATTCAGTGAATCATCGAATCTTTGAACGCACCTTGCGCTCCATGGTATTCCGTGGAGCATGCCTGTTTGAGTGTCATGAATACTTCAACCCTCCTCTTTCTTAATGATTGAAGAGGTGTTTGGTTTCTGAGCGCTGCTGGCCTTTACGGTCTAGCTCGTTCGTAATGCATTAGCATCCGCAATCGAACTTCGGATTGACTTGGCGTAATAGACTATTCGCTGAGGAATTCTAGTCTTCGGACTAGAGCCGGGTTGGGTTAAAGGAAGCTTCTAATCAGAATGTCTACATTTTAAGATTAGATCTC |
| Sequence of the isolate  (994 nucleotides)  NSF4/18 and EukR | >AGCGACGGGCGGTGTGTACAAAGGGCAGGGACGTAATCAACGCGATCTGATGAATCACGCTTACTAGGTATTCCTCGTTGAAGAACAATAATTGCAATGTTCTATCCCCATCACGACAGAGTTTCACAAGATTACCCATGCCTTCCGGCAAAGGTGGTAGACTCGCTGGCTCTGTCAGTGTAGCGCGCGTGCGGCCCAGAACATCTAAGGGCATCACAGACCTGTTATTGCCTCAAACTTCCATCAGCTAAACGCTGATAGTCCCTCTAAGAAGACAGCAGCTAGCCAAAGCCGGCTGGTCTATTTAGCAGGTTAAGGTCTCGTTCGTTATCGGAATTAACCAGACAAATCACTCCACCAACTAAGAACGGCCATGCACCACCAACCACAAGATCAAGAAAGAGCTATCAATCTGTCAATCCTTATTGTGTCTGGACCTGGTGAGTTTCCCCGTGTTGAGTCAAATTAAGCCGCAGGCTCCACACCTGGTGGTGCCCTTCCGTCAATTCCTTTAAGTTTCAGCCTTGCGACCATACTCCCCCCCGAACCTCATTTAAAGATTTCTCTTCGGGTGCCGATACAGTCATTAAAAATCCTGTACCGATCCCCAATTGGCATAGTTTACAGAAGAGACTACAACGGTATCTAATCGTTTTCGATCCCCCTTCCTTCGTTCTTGATCAATGAAAACATCCTTGGCAAATGCTTTCGCAGTAGTTTGTCTTCCGGCAATCCAAGAATTTCACCTCTGACGACGGAATACAAATGCCCCCAACTATCCCTATTAATCATTACGGCGATCTCAGAAACCAACAAAATGGGAACGCGCGTCCTATTTTATTATTCCATGCTAATGTATTCGGGCAAAGGCcTGCTTTGAACACTCTAATTTTTTCAAAGTAAAAGTCCTGGTTTGCGACGACACCCAGTAAAGGACATCGCCGTTCACCAGGAGGTAAGGCTCCGTCAAACAAGTACACACCAAGAAGGCGGA |
| Sequence of the culture prior to the activity assay  (540 nucleotides)  ITS1Fngs and ITS4ngsUni | >GCGGAAGGATCATTAGTGAATATAGGACGTCCAACTTAACTTGGAGTCCGAACTCTCACTTTCTAACCCTGTGCACTTGTTTGGGATAGTAACTCTCGCAAGAGAGCGAACTCCTATTCACTTATAAACACAAAGTCTATGAATGTATTAAATTTTATAACAAAATAAAACTTTCAACAACGGATCTCTTGGCTCTCGCATCGATGAAGAACGCAGCGAAATGCGATAAGTAATGTGAATTGCAGAATTCAGTGAATCATCGAATCTTTGAACGCACCTTGCGCTCCATGGTATTCCGTGGAGCATGCCTGTTTGAGTGTCATGAATACTTCAACCCTCCTCTTTCTTAATGATTGAAGAGGTGTTTGGTTTCTGAGCGCTGCTGGCCTTTACGGTCTAGCTCGTTCGTAATGCATTAGCATCCGCAATCGAACTTCGGATTGACTTGGCGTAATAGACTATTCGCTGAGGAATTCTAGTCTTCGGACTAGAGCCGGGTTGGGTTAAAGGAAGCTTCTAATCAGAATGTCTACATTTTAA |
