# Supplementary material for: Polyethylene degradation and assimilation by the marine yeast Rhodotorula mucilaginosa
Source: ISME Commun. 2023 Jul 10;3:68. doi: 10.1038/s43705-023-00267-z (PMC10330194; doi:10.1038/s43705-023-00267-z)
Supplement: Supplementary file 4 — Table S2 [file 43705_2023_267_MOESM4_ESM.docx]

| Incubation | ^13^C PE added (µg) | pH | Total amount CO_2_ (nmol) | Δ ^13^F CO_2_ d^-1^ | Total excess ^13^C (nmol) | Total degradation (µg) | % degradation | % yr^-1^ |
| --- | --- | --- | --- | --- | --- | --- | --- | --- |
| UV ^13^C-PE + RM | 1837 | 7.83 | 57728 | 0.000345 | 97.84 | 1.47 | 0.08 | 5.93 |
| UV ^13^C-PE - RM | 1660 | 8.13 | 151697 | 0.000043 | 31.70 | 0.48 | 0.03 | 2.13 |
| ^13^C-PE + RM | 1823 | 7.74 | 48152 | 0.0000001 | 0.03 | 0.00 | 0.00 | 0.00 |
